# A single vaccination of nucleoside-modified Rabies mRNA vaccine induces prolonged highly protective immune responses in mice

**DOI:** 10.3389/fimmu.2022.1099991

**Published:** 2023-01-17

**Authors:** Shimeng Bai, Tianhan Yang, Cuisong Zhu, Meiqi Feng, Li Zhang, Ziling Zhang, Xiang Wang, Rui Yu, Xinghao Pan, Chen Zhao, Jianqing Xu, Xiaoyan Zhang

**Affiliations:** ^1^ Shanghai Public Health Clinical Center & Institutes of Biomedical Sciences, Fudan University, Shanghai, China; ^2^ Clinical Center of Biotherapy, Zhongshan Hospital, Fudan University, Shanghai, China

**Keywords:** rabies, mRNA vaccine, rabies virus glycoprotein, virus-neutralizing antibodies, challenge model

## Abstract

**Background:**

Rabies is a lethal zoonotic disease that kills approximately 60,000 people each year. Although inactivated rabies vaccines are available, multiple-dose regimensare recommended for pre-exposure prophylaxis or post-exposure prophylaxis,which cuts down the cost- and time-effectiveness, especially in low- and middle incomecountries.

**Methods:**

We developed a nucleoside-modified Rabies mRNA-lipid nanoparticle vaccine (RABV-G mRNA-LNP) encoding codon-optimized viral glycoprotein and assessed the immunogenicity and protective efficacy of this vaccine in mice comparing to a commercially available inactivated vaccine.

**Results:**

We first showed that, when evaluated in mice, a single vaccination of RABV-G mRNA with a moderate or high dose induces more potent humoral and T-cell immune responses than that elicited by three inoculations of the inactivated vaccine. Importantly, mice receiving a single immunization of RABV-G mRNA, even at low doses, showed full protection against the lethal rabies challenge. We further demonstrated that the humoral immune response induced by single RABV-G mRNA vaccination in mice could last for at least 25 weeks, while a two-dose strategy could extend the duration of the highly protective response to one year or even longer. In contrast, the three-dose regimen of inactivated vaccine failed to do so.

**Conclusion:**

Our study confirmed that it is worth developing a single-dose nucleoside-modified Rabies mRNA-LNP vaccine, which could confer much prolonged and more effective protection.

## Introduction

Rabies is a fatal zoonotic disease that claims about 60,000 lives annually (1). It is a neurological illness caused by the Rabies virus (RABV), a single-stranded non-segmented negative-sense virus belonging to the genus Lyssavirus, in the family Rhabdoviridae (2). The genome of RABV encodes only five proteins, including nucleoprotein (N), phosphoprotein (P), matrix protein (M), glycoprotein (G), and large polymerase protein (L) (2). The glycoprotein, located on the surface of rabies virions, is the major inducer of virus-neutralizing antibodies (VNA) against RABV infection (3, 4). Dog biting accounts for 95% of human infections, with the virus spreading through bites or scratches, usually in the saliva (5). After its replication in the muscle tissue, the virus could infect the peripheral nerves; when the invasion of the brain occurs, the mortality rate is 100% as soon as the clinical symptoms appear (6).

Nowadays, inactivated rabies vaccines are the most widely used. However, they required repeated injections or adjuvants to induce sufficient neutralizing antibody titers against infection. For pre-exposure prophylaxis (PrEP), a 2-dose PrEP schedule on day 0 and day 7 was needed to elicit virus-neutralizing antibody titers greater than 0.5 international units (IU) (7–10); for post-exposure prophylaxis (PEP), 4 to 5 shots are required (11). Given the high financial costs associated with repeated vaccinations and limited access to medical resources in developing countries, particularly in Asia and Africa, developing novel rabies vaccines effective with single-dose would be a desirable step for curing rabies.Recently, messenger RNA (mRNA)-based vaccines have shown great promise as a non-traditional vaccine platform against many infectious diseases. The realization of such a promise depends on the advancement of various methods that enhance the potency of mRNA vaccines, including modified nucleoside incorporation (12, 13)and coding sequence optimization (14). Non-replicating mRNA-based rabies vaccines with exclusively unmodified nucleosides have been developed (15, 16), mainly by CureVac AG (Tübingen, Germany). The mRNA vaccine platform of CureVac AG, the RNActive platform, has been founded onusing a complex sequence optimization algorithm to alter the GC content of the mRNA coding sequence, intending toincrease the protein translation efficacy (14). A major improvement of the mRNA vaccine is the employment of modified nucleosides as blocks for mRNA synthesis, which confer important beneficial effects, particularly the increased antigen expression due to the enhanced mRNA stability and the prevention of unnecessary activation of innate immunity (17, 18). The effectiveness of nucleoside-modified mRNA vaccines against virus-causing diseases has been demonstratedin numerous preclinical investigations (19–24), culminated in its use as a major vaccine typelicensed for human vaccination against SARS-CoV-2 (25, 26). Despite ongoing studies on Rabies mRNA vaccines, like CV7201 or CV7202 from the CureVac AG, which required a single high-dose or two doses to achieve protective virus-neutralizing titers (VNTs) in preclinical studies (16, 27) and phase 1 clinical trial (28), no Rabies mRNA vaccine has been licensed for human use. In addition, there is a lack of investigation of the efficacy in the induction of virus-neutralizing antibodies and antiviral protectivity of nucleoside-modified Rabies mRNA vaccine, and its dose dependency, in animal models.

This study mainly evaluated the immunogenicity of a single vaccination with nucleoside-modified RABV-G mRNA vaccine versus a commercially available inactivated vaccine in BALB/c mice. We demonstrated that the mRNA vaccine could induce higher VNA production in a dose-dependent manner to protect vaccinated mice against lethal rabies challenges and has a better T-cell response in the spleen than the commercially available inactivated vaccine. Moreover, we investigated the duration of the humoral immune response following a single vaccination and two immunization doses in mice.

## Methods

### Cells, viruses, vaccines, and animals

HEK293 cells (ATCC) were cultured in complete Dulbecco’s modified Eagle’s medium supplemented with 10% fetal bovine serum (BI, Utah, USA) and 2% penicillin and streptomycin (BI, Utah, USA), and maintained at 37°C in 5% CO_2_ incubator. Purified Vero cell culture vaccine (a rabies vaccine made from aGV strain for human use, freeze-dried, labeled potency is 1 dose ≥ 2.5 IU)) was kindly donated by Rongan Biological Co., Ltd (Ningbo, China) and was used as a positive control for the vaccine efficacy comparison. Wuxi Xin Lianxin Biotech co. LTD (Wuxi, China) kindly donated the CVS-11 rabies challenge virus, which was cultivated in BHK-21 cells and titrated in BALB/c mice. The CVS-11 strain has been approved as a challenge virus in RABV-neutralizing antibody tests (29). Female BALB/c mice (6–8 week-old, Specific pathogen-free) purchased from Suzhou Huachang Biological Co., Ltd were housed in the Shanghai Public Health Clinical Center (SPHCC) animal facility.

### mRNA and LNP preparation

The mRNA vaccine described here was based on our conventional optimized non-amplifying mRNA platform (CN202211129466.2 and CN 202211129212.0).

T7 RNA polymerase synthesized mRNA using the linearized plasmid (synthesized by GENEWIZ) encoding a codon-optimized Pitman-Moore (PM) strain glycoprotein (accession number AJ871962) as a template. The 3’ untranslated region (UTR) of the synthetic plasmid has 250 poly-A inserted at the end of it, eliminating the requirement for poly(A) tailing with poly(A) polymerase during *in vitro* transcription. 1-methylpseudourine-5’-triphosphate (Nanjing Synthgene Medical Technology Co., Ltd.) instead of UTP was used to generate mRNA incorporating a modified nucleoside. Then, the *in vitro* transcribed mRNAs were capped using the Vaccinia Capping System and an mRNA Cap 2’-O-methyltransferase (Novo protein Shanghai, China). The mRNA was precipitated overnight with 2.5 moles or more of LiCl at −20°C for 30 min, and then centrifuged at maximum speed; the mRNA pellets were washed with 70% ethanol and finally suspended with RNase-free water. The nucleoside-modified mRNAs were identified by agarose gel and stored frozen at −20°C until use.

Using the self-assembly method mentioned earlier (24), a mixture of nucleoside-modified RABV-G mRNA and LNP was made. An aqueous solution containing mRNA at acidic pH4.0 was rapidly mixed with a lipid mixture in ethanol. LNPs contained the ionizable lipid (Dlin-MC3-DMA) AVT (Shanghai) Pharmaceutical Tech Co., Ltd., di-stearoyl phosphatidylcholine (DSPC), cholesterol, and PEG-lipid (PEG2000-DMG) at a ratio of 50:10:38:2 mol/mol. The mRNA and lipid mixture percentage were 3:1. After being dialyzed in PBS, the LNP-encapsulated mRNA was filtered by a 0.22m microfilter, then stored at 4°C. The encapsulation efficiency was analyzed using a Quanti-T RiboGreen RNA Assay Kit (Thermo Fisher Scientific). The particle size was detected by dynamic light scattering (DLS), and the zeta potential of mRNA RABV-G LNPs was measured through a Zetasizer instrument.

### mRNA transfection and protein expression

According to the manufacturer’s instructions, the RABV-G mRNA was transfected into HEK293T cells with Lipofectamine 3000 Reagent (Life Technologies). Briefly, 1 μg mRNA in 25 μL Opti-MEM (GIBCO, Gaithersburg, MD, USA) was mixed with 2 μL Lipofectamine 3000 in 25 μL Opti-MEM for 5 min, and then the Lipofectamine 3000-mRNA complex mixture was immediately added to the 24-well plates. Cells were harvested after transfection for 24 h and were lysed in RIPA lysis buffer (Life Technologies) for 30 min on ice. The expression of RABV-G protein was detected by Western blot. Samples were mixed with 4×SDS buffer (Takara), and boiled for 5 minutes, then transferred to PVDF membrane following separation on a 10% polyacrylamide gel. The membrane was blocked with 5% non-fat milk dissolved in PBS containing 0.01% Tween at room temperature for two hours. The RABV-G (glycoprotein) protein was detected using primary antibody Rab-50 (Santa Cruz) for 1 h, followed by secondary peroxidase-conjugated goat anti-mouse IgG(H+L) (Yeasen) for 1 h. The protein signals were detected by the SuperSignal West Pico Plus chemiluminescent substrate (Thermo Fisher Scientific).

### Mouse immunization experiments

In this study, there were three distinct immunization protocols.

In the first immunization protocol, female BALB/c mice were divided into five groups (n = 5), and vaccinations were performed *via* intramuscular(I.M.) route by injecting into the thigh muscles of the two hind limbs, 0.3 μg, 1 μg, 3 μg, or 10 μg RABV-G mRNA-LNP in 100μl volume, with empty LNP as the negative control. For a prime-boost regimen schedule, the animals were vaccinated twice with the same vaccine at a 3-week interval.

In the second immunization protocol, mice (n = 5) were I.M. immunized with 10 μg of RABV-G mRNA-LNP, and I.M. boosted with 10 μg or 1 μg. Then the antibody titers were monitored until 53 weeks.

In the third immunization protocol, a total of 26 mice were I.M. immunized once with 0.3 μg, 1 μg, or 3 μg of RABV-G mRNA, or the negative control (empty-LNP). Furthermore, a licensed inactivated vaccine (the positive control) (n = 26) was injected intramuscularly three times on days 0, 7, and 21 with 100 μl (0.1 human dose). Then spleens of each group of four mice were collected to detect RABV-G-specific T-cell responses at 10 or 30 days. For the rabies virus challenge, 13 mice of each group were challenged with 20-fold MLD_50_ (50 μl per mouse) of rabies virus CVS-11 (challenge virus standard-11) *via* the I.M. route. Then the body weight was monitored daily to evaluate the survival rate. Mice were euthanized at seven days post-infection (dpi). The brain tissues of infected mice were collected for viral RNA loads, pathological examination, and detection of viral RNA expression in the brain (three mice per group). To assess the endurance of the humoral immune response, the remaining five mice in each vaccinated group were monitored for at least 25 weeks.

### ELISA

RABV-G-specific antibodies were detected by ELISA. The specific ELISA plates were coated with 100 ng/well of RABV-G protein (AtaGenix) overnight at 4°C and blocked with 5% skim milk in PBST at room temperature for two hours. After two washes in PBST, coated plates were sequentially incubated with 2-fold serially diluted mouse sera for 3 hours at room temperature, followed by HRP-conjugated anti-mouse IgG (1:5,000) (Yeasen) for 1 hour at room temperature. If analyzing the subclass of antibodies, biotin-conjugated goat anti-mouse IgG1 (1:5,000) or IgG2a (1:5,000) antibodies (Abcam) were added and incubated at room temperature for 1 hour and then incubated with SA-HRP (1:5,000) (Yeasen) for another 1 h at room temperature. Finally, after five PBST washes, the plates were incubated with the substrate OPD (Sigma) and followed by H_2_SO_4_ (1 M) to stop the reaction. As previously mentioned, we measured the absorbance at 490 nm with a Synergy Microplate Reader (Bio-Tek) (30). The ELISA endpoint titers were considered the highest serum dilution, with an absorbance over two times that of the negative control mice sera.

### Enzyme-linked immunospot assay

The manufacturer’s protocol (BD Bioscience) assessed T-cell responses with the mouse IFN-γ ELISpot assay set. In short, the spleens were harvested from vaccinated BALB/c mice at 10- or 30-days post-vaccination. The plates of PVDF membrane were pre-coated with anti-mouse IFN-γ antibodies (5 μg/ml) overnight at 4°C and then blocked for 2 hours at room temperature. Next, the 2×10^5^ viable isolated splenocytes were added to each well, followed by stimulation with the RABV-G peptide pool (Chinapeptides Co., Ltd) for 18–24 h at 37°C. Then the plate was incubated with biotin-labeled IFN-γ antibodies (2 μg/ml) for 1 h following addition with SA-HRP (1:100 dilution). Finally, the plate was washed in PBS and reacted with an AEC substrate reagent. Reactions were stopped with water until the spots could be clearly observed. The numbers of spots were read and analyzed with a Bio-spot plate reader (ChampSpot 437III, Beijing Sage Creation Science Co., Ltd).

### Flow cytometry assay

To assess antigen-specific T-cell immune responses, we isolated the mouse splenocytes and stimulated them with the RABV-G peptide pool (1 μg/ml of individual peptide, 1 ×10^6^ cells/well). Golgiplug (BD Biosciences) was mixed with the cells after 1 h and incubated for another 5 h. Then cells were harvested, washed in PBS, and stained with Amcyan Live Dead Kit or antibodies to surface markers, including CD3 (Percpcy5.5, Clone 17A2, BioLegend), CD4 (AF700, Clone RM4-5, BD Biosciences), and CD8 (FITC, Clone 53-6.7, BioLegend). Subsequently, the stained cells were fixed and permeabilized in permeabilizing buffer (BD Biosciences), followed by staining with antibodies to anti-IFN-γ (PE, Clone XMG1.2, BD Biosciences), anti-IL-2 (APC, Clone JES6-5H4, BioLegend), or anti-TNF-α (BV605, Clone MP6-XT22, BioLegend). Data were collected using BD FACSAria III flow cytometer (BD Biosciences) and analyzed using FlowJo 10.

### Virus-neutralization measurement

Virus-neutralization antibody titers were determined with the fluorescent-antibody virus-neutralization (FAVN) assay as previously described (31). Briefly, 3-fold serial dilutions of standard serum (0.5 IU/ml) and test serum samples (four replicates per sample) were prepared in 96-well plates and mixed with 100 TCID_50_ of CVS-11 (50 μl/well). They were then incubated at 37°C for 1 h in a 5% CO_2_ incubator. Next, 50 μl of suspension containing 210^4^ BHK-21 cells was added to the mixture and continued to incubate at 37°C for 48 h. Cells were first fixed with 80% acetone for 30 min at 4°C and then stained with FITC-labeled RABV-N antibody (Veterinary Research Institute, Changchun, China). Fluorescence was observed under ultraviolet microscopy, and the VNA titers were determined and normalized compared with the value of the standard serum.

### Viral RNA extraction and RT-PCR

According to the manufacturer’s guide, total RNA was extracted from the brains of infected mice with the RNA isolation kit (Direct-zol RNA Miniprep Plus kit, Zymo Research). First, the rabies virus was detected using the Reverse Transcription System (Promega) and then SYBR green-based real-time PCR (GoTaq qPCR Master Mix; Promega) through a Bioer real-time PCR system with the N protein-specific primers. The oligo primers used were:

F: 5’-AATGCGACGGTTATTGCTGC-3’; R: 5’-TGCCACGTCGGTCTTTGTTA-3’.

The steps of real-time RT-PCR were carried out as follows: 42°C for 15 min and 95°C for 2 min, 40 cycles of amplification at 95°C for 15 s and 60°C for 30 s.

### Histopathology and RNA *in Situ* hybridization

Histopathological lesions in the brains were detected through hematoxylin and eosin staining. Brains were first fixed in 4% (v/v) paraformaldehyde and then prepared into paraffin sections (4–5 μm). For analyzing viral RNA expression in brain sections by RNAscope *in situ* hybridization (ISH), the viral nucleoprotein (NP) RNA was used as the target RNA for its high conservation. The NP-specific RNAscope probe was V-RABV-gp4 (220268) from ACDBio. The RNA ISH assay was performed with the previously described RNAscope Multiplex Fluorescent Reagent Kit V2 kit (Advanced Cell Diagnostics, 323100) (32).

### Ethics statement

All animal experiments in this study were approved by the Institutional Animal Care and Use Committee (IACUC) of the Shanghai Public Health Clinical Center. All the animal studies were strictly conducted following the animal ethics guidelines.

### Statistical analysis

For all the analyses, *p* values were obtained from the Mann-Whitney test or One-way ANOVA followed by Tukey’s multiple comparisons test through the GraphPad Prism. If *p*<0.05, data were determined statistically significant (*p< 0.05; **p< 0.01; ***p< 0.001; ****p< 0.0001; ns, not significant). All of the graphs were generated with GraphPad Prism Version 9.4 software.

## Results

### Design and characterization of RABV-G mRNA vaccine

**Figure 1:**
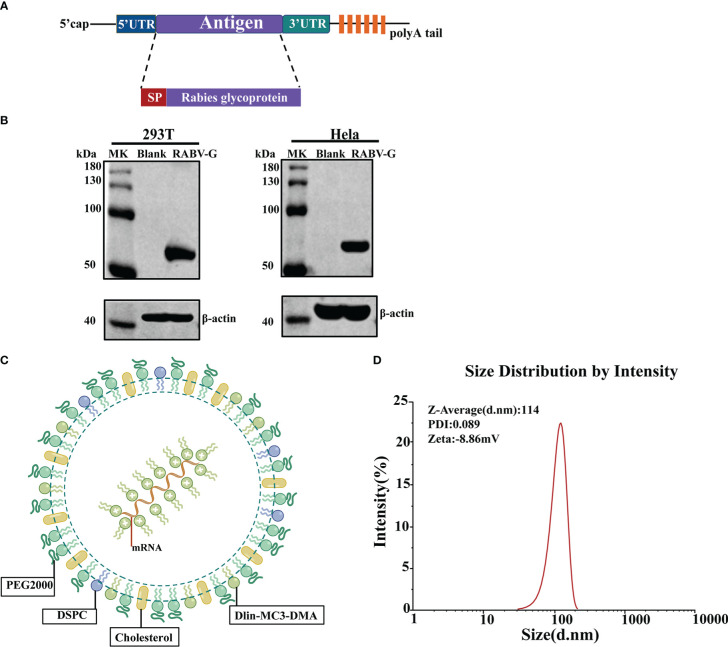
Design and Characterization of RABV-G mRNA Vaccine. **(A)** Schematic of RABV-G mRNA construct comprising a 5’cap, a 5’UTR, a signal peptide, an antigen (RABV-G from strain Pitman-Moore), a 3’UTR, and a 250 poly **(A)** tail. **(B)** RABV-G mRNA was transfected into HEK293T or Hela cells. RABV-G expression in the cell lysate at 24 h was analyzed by western blotting. **(C)** Schematic representation of the RABV-G mRNA packaged into LNPs. **(D)** Particle size graph of LNPs by dynamic light scattering.

To test our conventional optimized non-amplifying mRNA platform, we designed an mRNA vaccine that encodes the codon-optimized glycoprotein of the Rabies virus Pitman-Moore (PM) strain (**Figure 1A**). The synthesized RABV-G mRNA *in vitro* includes the modified nucleoside N1-methylpseudouridine to suppress innate immune sensing and enhance mRNA translation (21). Then we examined the RABV-G mRNA expression by transfecting HEK293T cells or Hela cells. Western blot demonstrated that RABV-G protein could be expressed effectively and with the right size (67 kDa) in transfected cells (**Figure 1B**). To improve the mRNA expression *in vivo*, we encapsulated the RABV-G mRNA in lipid nanoparticles (LNPs). The LNPs had a ratio of 50:10:38:2 for the four lipids, Dlin-MC3-DMA, DSPC, cholesterol, and PEG2000-DMG (**Figure 1C**). Furthermore, dynamic light scattering analysis showed that the average particle size of LNPs in PBS was 114 nm, with a PDI of 0.089 (**Figure 1D**). Then a Zeta potential measurement showed a potential of −8.86 mV in PBS (**Figure 1D**).

### Immunogenicity evaluation of various doses of nucleoside-modified RABV-G mRNA vaccine

**Figure 2:**
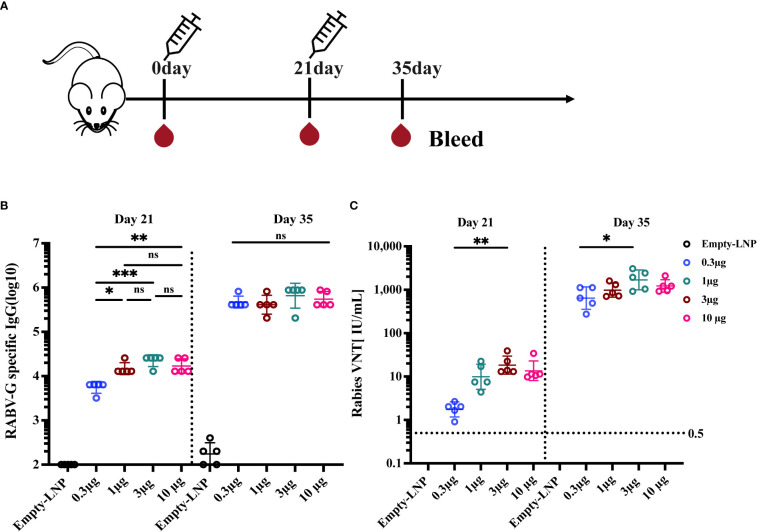
Immunogenicity evaluation of various doses of RABV-G mRNA Vaccine. Female BALB/c mice (n=5) were immunized with 0.3 μg, 1 μg, 3 μg, and 10 μg RABV-G mRNA or empty-LNP as a negative control with a prime-boost regimen at a 3-week interval **(A)**. Serum was collected on days 21 and 35 for analysis. RABV-G-specific IgG and virus-neutralizing antibody titers were measured as shown in **(B)** and **(C)**, respectively. The dashed line at 0.5 IU/ml represents a protective titer for Rabies VNTS. Each point represents an individual mouse. Titer data were GMT + geometric standard deviation (GSD). Comparisons among experimental groups were determined using One-way ANOVA followed by Tukey’s multiple comparisons tests (*p< 0.05; **p< 0.01; ***p< 0.001; ns, not significant).

The immune responses induced by our RABV-G mRNA vaccine were analyzed in BALB/c mice. First, we assessed the effects of different vaccination dosages on the immunogenicity of vaccines. Female BALB/c mice were divided into five groups (n = 5) and intramuscularly injected with 0.3 μg, 1 μg, 3 μg, and 10 μg RABV-G mRNA or the equivalent amount of empty-LNP without mRNA as a negative control, with a prime-boost regimen at 3-week intervals (**Figure 2A**). Serum samples were harvested on days 21 and 35 to measure RABV-G-specific IgG and virus-neutralizing titers. The virus-neutralization antibody titers (VNTs) in different weeks post-immunization were determined by the FAVN method as shown in the method. Mice receiving 1 μg, 3 μg, or 10 μg doses of the RABV-G mRNA produced high endpoint binding titers on day 21 after the initial vaccination, whereas 0.3 μg of RABV-G mRNA induced the lowest antibody titers or VNTs in all immunized groups (**Figures 2B, 2C** *p*< 0.05, *p*< 0.01). Two weeks after the second immunization, all four separate doses (0.3 μg, 1 μg, 3 μg, and 10 μg) successfully induced the RABV-G binding antibody titers, with a geometric mean titer (GMT) of 470507, 409600, 654324, and 547615, respectively, and no statistically significant differences across any of the dosages (**Figure 2B**). Notably, all inoculated mice in each group developed VNTs above 0.5 IU/ml, whether 21 days after the initial immunization or two weeks after the boost (**Figure 2C**). Moreover, a dose-dependent increase in neutralization titer was observed; the VNTs at 21 days post-vaccination ranged from 1.75 to 18.3 IU/ml (**Figure 2C**, *p*< 0.05), while the VNTs increased by 100-fold or more following a second injection, with an average of 642, 979, 1689, and 1221 IU/ml (as in 0.3 μg, 1 μg, 3 μg, and 10 μg groups; **Figure 2C**, *p*<0.01). Interestingly, it showed that the 3 μg group, rather than the ​10 μg one, produced the highest VNTs among these doses.

### A single RABV-G mRNA vaccination elicits high and Th-1 biased humoral immune response in mice

**Figure 3:**
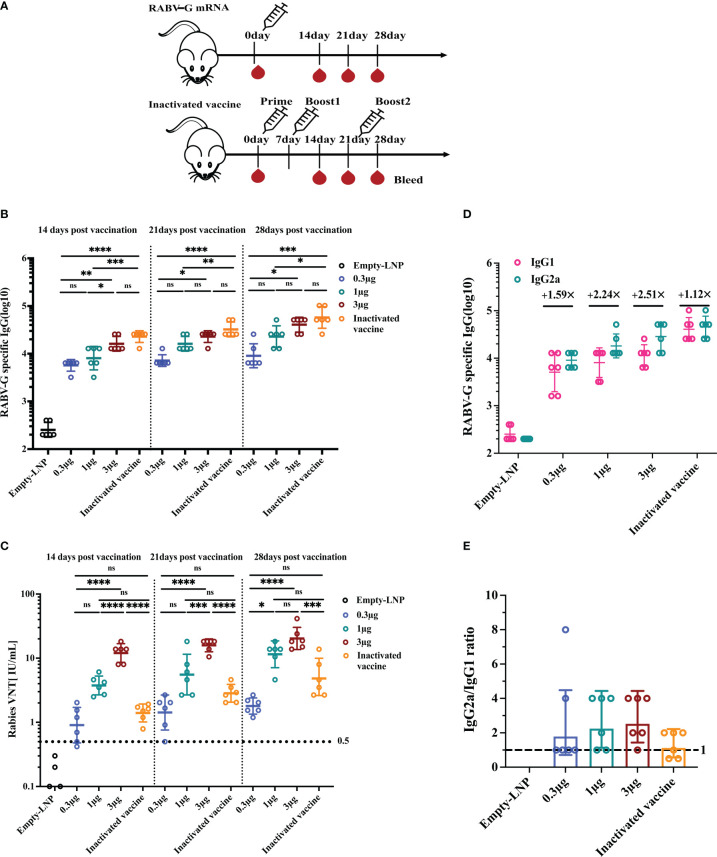
Humoral immune response upon single vaccination with escalating doses of RABV-G mRNA compared to three injections of inactivated vaccine. Female BALB/c mice (n = 6) were inoculated with a single intramuscular injection of RABV-G mRNA at various doses (0.3g, 1g, and 3g) or an empty-LNP **(A)**. As a positive control, a licensed inactivated vaccine group (n = 6) was injected three times on days 0, 7, and 21 with 100l (0.1 human dose) *via* the intramuscularly (I.M.) route. Serum was collected on days 14, 21, and 28 to detect specific antibody responses **(B)** and virus-neutralizing antibody titers **(C)**. Serum levels of IgG1 and IgG2a antibodies specific for RABV-G **(D)** and IgG2a/IgG1 ratios **(E)** were evaluated by ELISA. Comparisons among experimental groups were determined using One-way ANOVA followed by Tukey’s multiple comparisons tests (*p< 0.05; **p< 0.01; ***p< 0.001; ****p< 0.0001; ns, not significant).

Having demonstrated that each dosage could induce a viral neutralization titer (VNT) higher than 0.5 IU/ml, considered to be protective in humans, dogs, and cats, we determined the immune responses of a single vaccination with various doses. Groups of mice (n = 6) were immunized I.M. once with a low dose of 0.3 μg, a moderate dose of 1 μg, a high dose of 3 μg, or an empty-LNP (**Figure 3A**). As a positive control, a licensed inactivated vaccine group was injected I.M. three times on days 0, 7, and 21 with 100 μl (0.1 human dose; according to the immunization procedure of the donated inactivated vaccine; **Figure 3A**). By ELISA, serum samples obtained 2, 3, and 4 weeks post-vaccination were evaluated for RABV-G-specific IgG and IgG subtypes. All three dosages induced potent RABV-G-specific antibody and VNA responses after a single immunization. Binding affinity for the RABV-G and virus-neutralizing titers became detectable two weeks after immunization and continued to rise between 2 and 4 weeks. As a result, at week 2, the geometric mean endpoint titers of RABV-G-specific IgG in mice immunized with the high dose rose to 16127. They were significantly higher than those observed in mice immunized with the low dose (5701.5; **Figure 3B**, *p*<0.001, **Supplementary Figure 1A**). By week four after immunization, compared to the inactivated vaccine group (57470.1), the high-dose group had an average binding endpoint titer of 40637.5. In contrast, those moderate-dose and low-dose animals had respective values of 22807 and 9050.97 (**Figure 3B**, **Supplementary Figure 1A**). The high-dose group elicited the highest VNTs of 20.3 IU/mL for virus-neutralizing activity at week 4. They were 10-, 1.7-, and 5-fold higher than those observed in the low-dose group (*p*<0.0001), moderate-dose one (*p*>0.05), and the inactivated vaccine group (*p*<0.001), respectively (**Figure 3C**, **Supplementary Figure 1B**). These results indicate that the three doses are all immunogenic, but the high-dose group (3 μg) was superior in inducing binding and neutralizing antibody responses in BALB/c mice. Additionally, all three doses elicited IgG2a and IgG1 subclass RABV-G-specific antibodies in sera collected at week four post-prime (**Figure 3D**). The assessment revealed an average IgG2a/IgG1 ratio above one across all groups, indicating a Th1-biased response (**Figure 3E**).

### RABV-G mRNA vaccines effectively elicit an antigen-specific T-cell immune response

**Figure 4:**
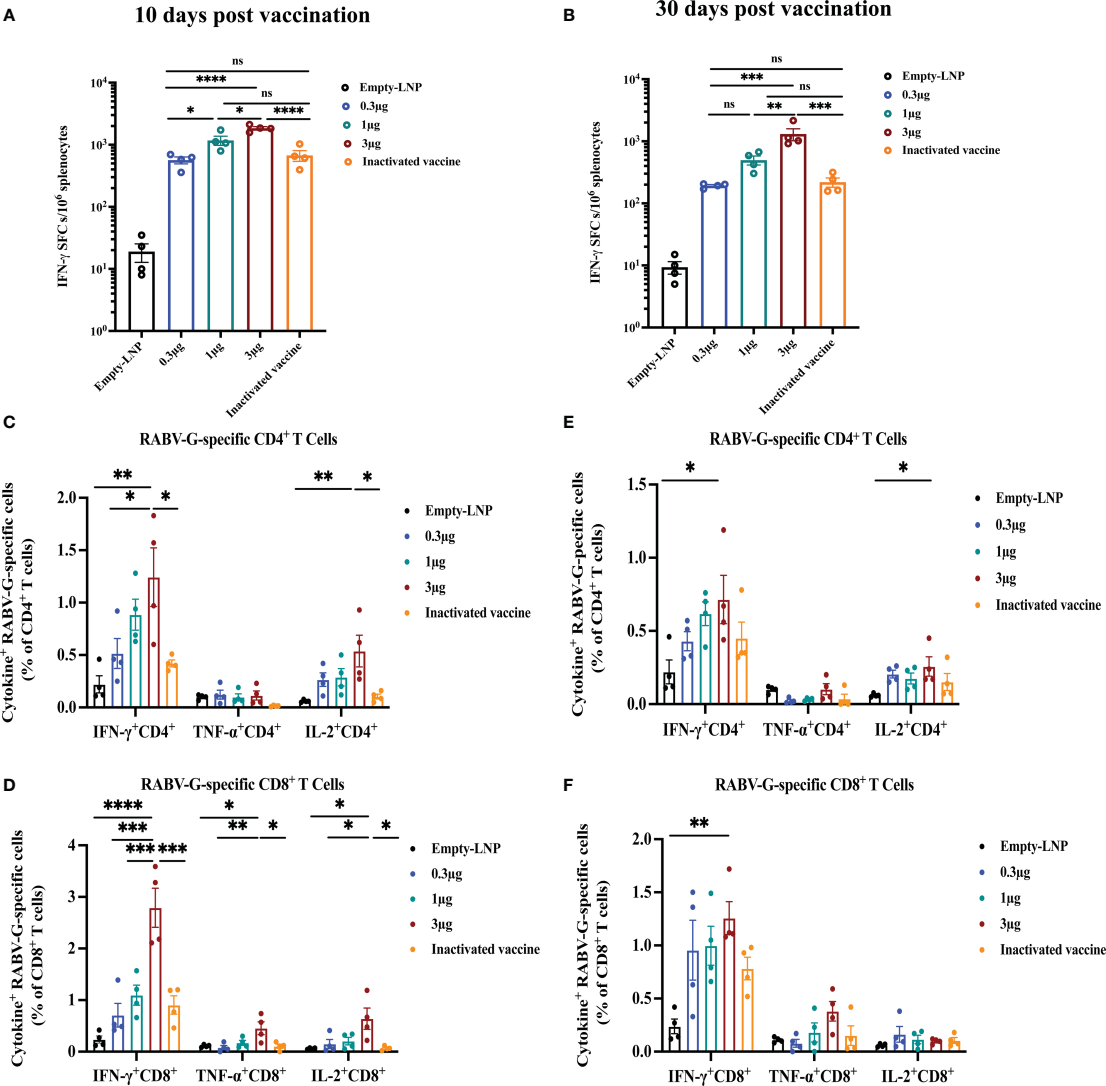
T-cell immune responses in vaccinated mice. An ELISPOT experiment was conducted to determine the ability of splenocytes to release IFN- after re-stimulation with RABV-G peptide pools at ten days **(A)** or 30 days post-vaccination **(B)**. Then RABV-G-specific CD4+ and CD8+ T cells producing IFN-γ, TNF-α, and IL-2 were measured by flow cytometry at ten days **(C, D)** or 30 days **(E, F)**. The data are shown as the number of IFNγ spot forming cells (SFC)/10^6^ splenocytes. ELISpot counts were represented as mean + SEM (standard error of the mean). Comparisons among experimental groups were determined using one-way ANOVA followed by Tukey’s multiple comparisons tests (*p< 0.05; **p< 0.01; ***p< 0.001; ****p< 0.0001; ns, not significant).

To evaluate the cellular responses induced by RABV-G mRNA with various doses (0.3 μg, 1 μg, 3 μg) or the licensed inactivated vaccine as a positive control, enzyme-linked immunospot (ELISPOT) and intracellular cytokine staining (ICS) assays were carried out at ten days or 30 days post-immunization. The splenocytes isolated from vaccinated mice (n = 4) were re-stimulated with a pooled library of RABV-G peptides *in vitro*. Ten days post-vaccination, ELISPOT assays demonstrated that T cells secreting gamma interferon (IFN-γ) from immunized mice of the high dose group (3 μg; Mean = 1844) were significantly more numerous than those from the inactivated vaccine group (the group received two injections at day 10; Mean = 641; **Figure 4A**, *p*<0.0001), while the specific T cells of mice in the low dose (0.3 μg; Mean = 564) or the moderate dose (1 μg; Mean = 1175) group were comparable to the mice receiving the licensed inactivated vaccine (**Figure 4A**). Moreover, flow cytometric analysis also showed that RABV-G-specific polyfunctional CD4+ and CD8+T cells secreting interferon γ (IFN-γ) and interleukin-2 (IL-2) were significantly elevated in RABV-G mRNA immunized mice (especially the high dose group) compared to inactivated vaccine-treated animals (mean of 1.2% for IFN-γ expressing CD4+ T cells after the high dose RABV-G mRNA and 0.42% after inactivated vaccine immunization) (**Figure 4C**, **Supplementary Figure 2**). In contrast, frequencies of antigen-specific tumor necrosis factor-alpha (TNF-α) were low across the groups (**Figures 4C, D**, **Supplementary Figure 2**).

Additionally, we further examined RABV-G mRNA induced cellular immune at 30 days post-vaccination. Compared to the inactivated vaccine-treated group (the one that received three injections on day 30), the stimulated splenocytes in the high-dose group produced a higher population of CD4+ and CD8+ cytokine IFN-γ- and TNF-α- expressing cells (**Figures 4E, F**). The frequencies of antigen-specific polyfunctional CD4+ and CD8+T cells were similar in the other two groups (the moderate and low doses; **Figures 4E, F**). ELISPOT analysis also revealed significant induction of IFN-γ in the splenocytes of RABV-G mRNA vaccinated mice in the high dose group (Mean = 1307) than in the inactivated vaccine one (Mean = 220) (**Figure 4B**, *p*<0.001). Our results demonstrated that the RABV-G mRNA vaccine could effectively activate RABV-G-specific antigen T-cell responses in addition to humoral immune responses, the frequencies of antigen-specific T-cells were dose-dependent, and better induction of T cells by our RABV-G mRNA vaccine compared to the inactivated vaccine.

### RABV-G mRNA vaccines confer complete protection against the rabies virus

**Figure 5:**
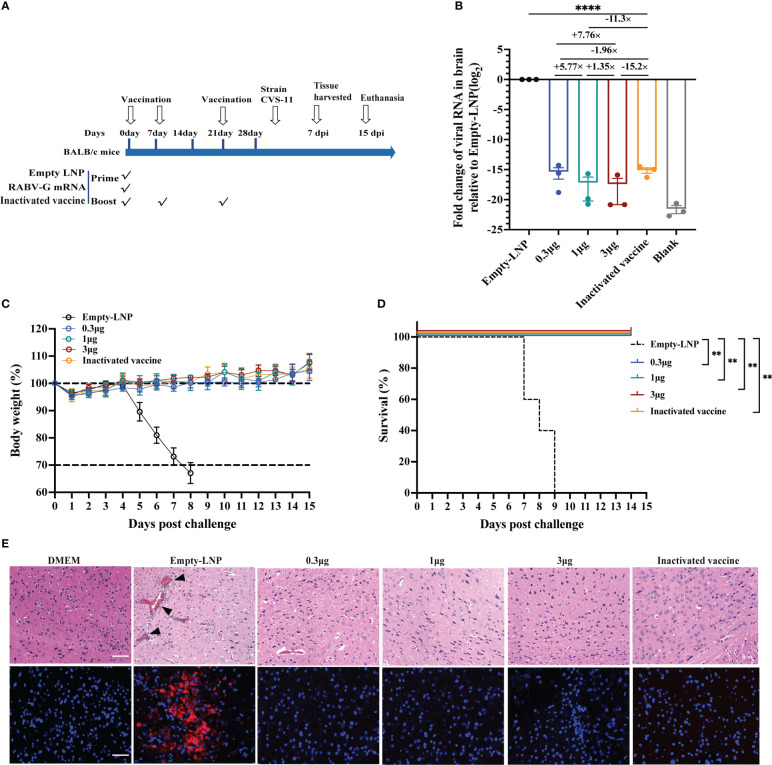
Protection of RABV-G mRNA in BALB/c mice against rabies virus challenge. Groups of female BALB/c mice (n = 13) received a single dose of RABV-G mRNA or three doses of inactivated vaccine or empty-LNP via the I.M. route. Four weeks post initial vaccination, mice were I.M. challenged with 20MLD50 of CVS-11 virus in a total volume of 50µl. **(A)** Mice immunization and challenge schedule. The black hollow arrows indicate the time of vaccination and the virus challenge. **(B)** Relative messenger RNA (mRNA) expression of viral N protein (log2 fold change from Empty-LNP group) on day seven post-infection.Comparations among experimental groups were determined using one-way ANOVA followed by Tukey’s multiple comparisons tests (****p< 0.0001). **(C)** The body weight of challenged mice was monitored daily and is shown as the mean + SEM (n = 5). As soon as animals lost 25% of their initial body weight (dotted line), they were sacrificed. **(D)** A Kaplan-Meyer analysis illustrates the survival curves during a 15-day observation period. Bars represent the mean and SEM (n = 5/group, **p <0.01). **(E)** H&E staining and RNAscope in situ hybridization (ISH) assay of Brain tissues from DMEM group mice or infected mice (n = 3/group). At 7 d.p.i., sagittal sections of the mouse brain were cut and stained with H&E, histopathological analysis was performed, and the representative histological changes (scale bars, 50 µm) are presented. Black triangles indicate pathological changes, including inflammatory cuffs of blood vessels (perivascular cuffing) and/or intravascular coagulations. Representative images of ISH showed virus NP expression in the brain. Each red dot represents a single NP RNA molecule, with nuclei counterstained by DAPI.

To explore the *in vivo* protection efficacy of the RABV-G mRNA vaccine against lethal rabies virus challenge, BALB/c mice (n = 13) received one immunization with three dosages 0.3 μg, 1 μg, and 3 μg) of RABV-G mRNA or empty-LNP *via* I.M. route, and positive control mice (n = 13) were vaccinated three times intramuscularly with a 0.1 human dose of the inactivated vaccine (**Figure 5A**). All mice were challenged with 20-fold MLD_50_ of rabies virus CVS-11 (challenge virus standard-11) *via* the I.M. route on day 30 (**Figure 5A**). We monitored the body weight of each mouse daily for 15 days post-infection. We found that body weight dropped at 1 dpi compared to the empty-LNP group but increased more rapidly during the following days (**Figure 5C**). There were no significant differences in body weight among each vaccinated group (**Figure 5C**). Moreover, all vaccination groups survived post-infection (**Figure 5D**). Three mice of each group were euthanized at 7 dpi, brain tissues of infected mice were collected for viral RNA loads and histopathology, and RNAscope analyzed the viral expression *in situ* hybridization (ISH; **Figure 5E** down). Half of the brain was tested for rabies virus replication by quantitative RT-PCR of RNA encoding the rabies nucleoprotein (N protein). As expected, all mice in the empty-LNP group had abundant rabies virus RNAs (**Figure 5B**). In contrast, most animals in the vaccination groups had extremely low but detectable levels (**Figure 5B**). Compared to the positive control animals, the average brain viral RNA levels for high-dose, moderate-dose, and low-dose groups were approximately 15.2-fold, 11.3-fold, and 1.96-fold lower, respectively. There were significantly (*p* < 0.0001) lower rabies virus RNA levels in the brain of all four immunized groups compared with the empty-LNP group (**Figure 5B**). Histopathological analyses revealed intravascular coagulation and perivascular cuffing in the empty-LNP group (**Figure 5E** top). In contrast, no lesion changes were observed in the high-dose, moderate-dose, or positive control groups, and only a few intravascular coagulations in the brain of the low-dose group (**Figure 5E**). Similarly, dot signals of viral RNAs were detected in the brains of empty-LNP mice using RNAScope but not in the vaccinated groups (**Figure 5E**). These results showed that even at a low dose, single immunization with our nucleoside-modified RABV-G mRNA vaccine afforded complete protection against the lethal challenge of rabies virus in mice.

### Immune response kinetics following vaccination of RABV-G mRNA

**Figure 6:**
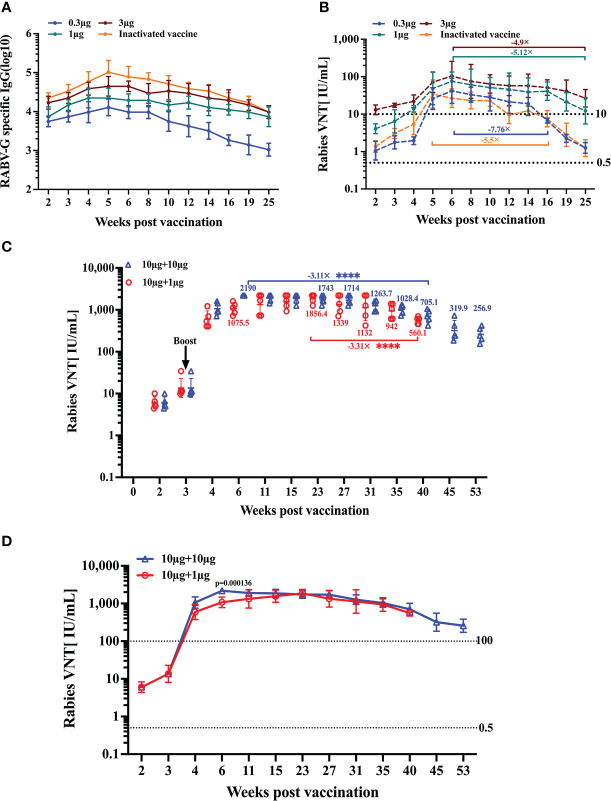
Duration of immune response induced by RABV-G mRNA. The durability of the antibody response was analyzed using **(A)** ELISA of total IgG and **(B)** virus-neutralizing antibody titers up to 25 weeks after a single-dose immunization or three injections of inactivated vaccine. Kinetic of virus-neutralizing antibody from two I.M. immunizations with different dosages in 3-week intervals; the initial vaccination was 10 μg, and the booster was 10 μg or 1 μg **(C, D)**. Titer data are shown as GMT + GSD. Comparisons among experimental groups were determined using one-way ANOVA followed by Tukey’s multiple comparisons tests ****p< 0.0001.

Prophylactic immunization must produce long-lasting protection. First, we assessed the endurance of the humoral immune response following a single vaccination. The remaining BALB/c mice (n = 5) of each group were bled from the time of immunization until 25 weeks after that. We found that the peak RABV-G-specific antibody level in the inactivated vaccine group was higher than in other groups (**Figure 6A**). However, the peak neutralizing antibody titers in the high-dose group (3 μg) were at average higher than those in the positive control group. (**Figure 6B**). (**Figure 6B**). Notably, the VNTs of the RABV-G mRNA groups peaked six weeks after immunization and remained stable during the 6–14 week period (**Figure 6B**). Furthermore, we observed a dramatic fall in VNTs to 6.8 and 8.5 IU/mL in both the low dosage and positive groups at week 16, with a decrease of 7.76-fold and 5.5-fold compared to their peak VNTs (**Figure 6B**). At week 25, the VNA titers in the moderate and high dose groups decreased by 5.12-fold and 4.9-fold from their peak VNTs, but remained higher than 10 IU/mL, whereas the other groups’ levels dropped to 1.3 IU/mL, a decrease of nearly 40-fold.

Following a single dose of vaccination, effective neutralizing antibody responses persisted for at least 25 weeks; we wondered whether a second booster immunization would help the VNA response sustain at higher titers for longer. Thus, we evaluated the kinetics of induced humoral response following two vaccinations. Mice (n = 5) received two I.M. immunizations with different dosages at 3-week intervals. The initial vaccination was 10 μg, and the booster was 10 μg or 1 μg, respectively. Monitoring the antibody titers for 53 weeks in a group receiving two I.M. immunizations of 10 μg, we could demonstrate that neutralizing titers stabilized at a level of about 1000–2000 IU/ml for 4–35 weeks. In contrast, at week 40 post-initial immunization, the VNT decreased by 3.11-fold from their peak of 2190 IU/mL (**Figures 6C, D**). While in the 1 μg boost group, neutralizing titers rose to 594.8 IU/mL at week 4, peaked at 23 weeks, then, at week 40, dropped to 560.1 IU/mL, a decrease of 3.31-fold from the peak VNTs (**Figures 6C, D**).

These data suggest that a single immunization with RABV-G mRNA (even a low dose) through the I.M. route can induce a strong and durable antibody response for at least half a year. Furthermore, we observed that pronounced boosting effects were achieved through a second immunization regardless of whether it was a high or low-dose booster. High-neutralizing antibodies could be maintained for one year or much longer.

## Discussion

Rabies is an infectious mortality disease that occurs worldwide. Current commercial inactivated vaccines have relatively poor immunogenicity and require repeated injections. In recent years, numerous studies have been carried out to develop novel, safe, and effective rabies vaccines, such as recombinant virus-vector vaccines (33–38), DNA and RNA-based vaccines (15, 16, 27, 28), live attenuated rabies vaccines (39), and protein vaccines (40). In this study, we mainly demonstrated that a single immunization of nucleoside-modified RABV-G mRNA vaccine induced a more potent immunogenic response than commercial inactivated rabies vaccines, providing complete protection against lethal rabies virus challenge, and induced durable NAb responses in BALB/c mice.

We first found that the RABV-G mRNA vaccine with a dose of 0.3 μg could elicit strong humoral immune responses following a two-dose immunization regimen, with neutralizing antibody titers of 642 IU/ml. In comparison, in a previous study, a dose of 0.5 μg LNP formulated nucleoside-unmodified RABV-G mRNA induced a median VNT of 650 IU/ml after two injections in murine models (16). Furthermore, a dose-dependent increase in neutralization titer was observed with a prime-boost regimen. We further probed the immune response of a single vaccination. We demonstrated that a single administration of RABV-G mRNA at doses of 0.3 μg, 1 μg, and 3 μg elicited VNTs of 1.8, 11.5, and 20.3 IU/ml, respectively, at four weeks post-vaccination. Consistent with the VNTs, our results showed that the single dose of RABV-G mRNA-LNP conferred complete protection against the rabies virus of CVS-11 infection *in vivo*, even at a low dose of 0.3 μg in mice. Then the VNTs were monitored for the following 25 weeks, and it was apparent that RABV-G mRNA peaked six weeks after immunization and remained at high levels in the ensuing period. The inactivated vaccine group dropped to 1.3 IU/mL at week 25, but the VNTs in the moderate and high dose groups remained higher than 10 IU/mL. Surprisingly, we found that the inactivated vaccine has the highest IgG-specific levels but the lowest VNTs. One possible reason may be the disruption of the heterogeneous structure of RABV-G on the virion surface of the inactivated vaccine. This structural heterogeneity may have an impact on the production of neutralizing antibodies that frequently target quaternary epitopes and may result in the short duration of the postvaccination immunological response (41).

It has been shown that T-cell immunity responses—particularly the Th1 immune response—are essential for eliminating RABV viruses from the central nervous system (42, 43). In our study, the single RABV-G mRNA vaccination strongly elicited Th1-biased responses in BALB/c mice. In addition, it effectively activated obvious RABV-G antigen-specific CD4+ and CD8+ T-cell responses, in line with the findings of previous mRNA vaccines (44, 45). Thus, the help of RABV-G antigen-specific T-cell responses may be one of the possible explanations for the sustained VNTs after a single RABV-G mRNA vaccination.

Recently, the recombinant virus-vector vaccine, like ChAd155-RG, has reported that a single vaccination elicited protective efficacy against rabies challenges in animal models (34). Compared to the single immunization of ChAd155-RG, which induced a peak VNT of 30 IU/mL (at a high dose of 10^8^ VP) in outbred mice, our single high-dose group produced a higher peak VNT of 100 IU/mL in BALB/c mice. It was comparable with ChAdOx1 or ChAdOx2 adenovirus-vectored rabies vaccines (at a high dose of 1 × 10^8^ IU) (36) (both peak VNTs around 100 IU/mL).

The rabies virus is usually transmitted through dog bites in developing countries, like Asia and Africa, and children are at high risk of exposure. Therefore, the rabies vaccine should be included in childhood immunization programs. The previous research demonstrated that using rabies vaccines for vaccination programs in children could produce recall responses rapidly (46). In this study, we also observed the kinetics of induction of VNA by RABV-G mRNA-LNP following two inoculations in mice; the durability of the serum VNA response after two vaccinations remained stable with a high VNT of 1,000 IU/ml up to almost one year. Therefore, the RABV-G mRNA may be one of the candidates for Children’s vaccination programs. Moreover, it also could be combined with adenovirus-vectored vaccines in a prime-boost regimen to circumvent the problem of pre-existing anti-vector antibodies.

According to the phase 1 clinical trials conducted by CureVac AG, CV7201 generated virus-neutralizing antibodies above the 0.5 IU/mL threshold in up to 83% of volunteers and needed repeated injections to meet the threshold (15). Then CV7202 could induce modest protective virus-neutralizing antibodies with two immunizations, whereas a single dose failed to elicit VNTs over 0.5 IU/mL. Likewise, the high doses were not well tolerated, and the trial had to be temporarily stopped (28). Similar disappointing results were also demonstrated in their non-nucleoside-modified SARS-CoV-2 mRNA vaccines (47). Nevertheless, the nucleoside-modified SARS-CoV mRNA vaccine exhibited good tolerance at higher dosages and superior immunogenicity (25, 26). In our study, we employed the powerful nucleoside-modified mRNA-LNP vaccine platform, which has produced effective clinical vaccine candidates against SARS-CoV-2 (25). Therefore, we hypothesized that our mRNA vaccine candidate RABV-G could produce excellent immune protective efficacy *in vivo* only with a single dosage due to the adoption of different vaccine platforms.

There were also several limitations in our study that warrant future investigation. Firstly, we have not had a chance to compare our vaccine with mRNA vaccines generated from unmodified nucleotides, which would allow us to determine the potential benefits associated with switching from unmodified to modified nucleotides. However, the advantage of unmodified nucleotides has been clearly demonstrated by previous studies, and it is tempting to speculate that such an advantage is also applicable to the Rabies mRNA vaccine, underlying the ability of RABV-G mRNA-LNP to induce robust neutralizing titers with a single low dose. Secondly, it is worth notifying there exists uncertainty about the translation of vaccination success in mouse study to human use. Further investigation of vaccine efficacy in a more clinically relevant animal model, like non-human primates, is necessary for deriving the vaccination regimen optimal for human use in terms of both safety and protective efficiency. Nevertheless, our results provide proof-of-concept evidence supporting the feasibility of developing a nucleoside-modified Rabies mRNA vaccine capable of affording safe, effective, and durable protection with a single dose.

## Data availability statement

The datasets presented in this study can be found in online repositories. The names of the repository/repositories and accession number(s) can be found in the article/**Supplementary Material**.

## Author contributions

SB and TY performed experiments and analyzed data. CZ, MF, LZ, ZZ, XW, RY, and XP conducted the mice sample collection, Elisa, or Virus-neutralization measurement. SB wrote the manuscript. XZ, JX, and CZ organized the study, guided experiments, and revised the manuscript. All authors contributed to the article and approved the submitted version.
