# Corrigendum: A single vaccination of nucleoside-modified Rabies mRNA vaccine induces prolonged highly protective immune responses in mice

**DOI:** 10.3389/fimmu.2023.1146466

**Published:** 2023-01-26

**Authors:** Shimeng Bai, Tianhan Yang, Cuisong Zhu, Meiqi Feng, Li Zhang, Ziling Zhang, Xiang Wang, Rui Yu, Xinghao Pan, Chen Zhao, Jianqing Xu, Xiaoyan Zhang

**Affiliations:** ^1^ Shanghai Public Health Clinical Center & Institutes of Biomedical Sciences, Fudan University, Shanghai, China; ^2^ Clinical Center of Biotherapy, Zhongshan Hospital, Fudan University, Shanghai, China

**Keywords:** rabies, mRNA vaccine, rabies virus glycoprotein, virus-neutralizing antibodies, challenge model

In the published article, there were three errors in the legend for Figure 5. In the starting statement, “…mice were I.M. challenged with 20MLD50 (50l per mouse) of the CVS-11 virus…” should be corrected to “… mice were I.M. challenged with 20MLD50 of CVS-11 virus in a volume of 50μL…”. There were two errors in Figure 5D: the statement of “n=3/group, **p <0.01” should be corrected to “n = 5/group, **p <0.01”, whereas the statement of “Comparations among experimental groups were determined using one-way ANOVA followed by Tukey’s multiple comparisons tests (****p< 0.0001)” was misplaced and should be moved to Figure 5B.

The corrected legend appears as below.

“Protection of RABV-G mRNA in BALB/c mice against rabies virus challenge. Groups of female BALB/c mice (n = 13) received a single dose of RABV-G mRNA or three doses of inactivated vaccine or empty-LNP *via* the I.M. route. Four weeks post initial vaccination, mice were I.M. challenged with 20MLD50 of CVS-11 virus in a total volume of 50μl. (A) Mice immunization and challenge schedule. The black hollow arrows indicate the time of vaccination and the virus challenge. (B) Relative messenger RNA (mRNA) expression of viral N protein (log2 fold change from Empty-LNP group) on day seven post-infection. Comparations among experimental groups were determined using one-way ANOVA followed by Tukey’s multiple comparisons tests (****p< 0.0001). (C) The body weight of challenged mice was monitored daily and is shown as the mean + SEM (n = 5). As soon as animals lost 25% of their initial body weight (dotted line), they were sacrificed. (D) A Kaplan-Meyer analysis illustrates the survival curves during a 15-day observation period. Bars represent the mean and SEM (n = 5/group, **p <0.01). (E) H&E staining and RNAscope *in situ* hybridization (ISH) assay of Brain tissues from DMEM group mice or infected mice (n = 3/group). At 7 d.p.i., sagittal sections of the mouse brain were cut and stained with H&E, histopathological analysis was performed, and the representative histological changes (scale bars, 50 μm) are presented. Black triangles indicate pathological changes, including inflammatory cuffs of blood vessels (perivascular cuffing) and/or intravascular coagulations. Representative images of ISH showed virus NP expression in the brain. Each red dot represents a single NP RNA molecule, with nuclei counterstained by DAPI.”

The authors apologize for these errors and state that this does not change the scientific conclusions of the article in any way. The original article has been updated.

