# Supplementary material for: A single vaccination of nucleoside-modified Rabies mRNA vaccine induces prolonged highly protective immune responses in mice
Source: Front Immunol. 2023 Jan 17;13:1099991. doi: 10.3389/fimmu.2022.1099991 (PMC9907168; doi:10.3389/fimmu.2022.1099991)
Supplement: Supplementary file 1 [file DataSheet_1.docx]

Supplementary Material

**Supplementary Data**

**Supplementary Figure 1. RABV-G specific antibody and virus-neutralizing antibody titers of each vaccinated group.** RABV-G-specific binding antibody titers (A) and neutralizing antibody titers (B) as assessed on days 14, 21, and 28. Titer data are shown as GMT + GSD.

**Supplementary Figure 2. Gating strategies for intracellular cytokine staining.** Flow cytometric gating strategy for investigating T-cell responses in RABV-G mRNA or inactivated vaccine-immunized mice is presented in Fig.4.


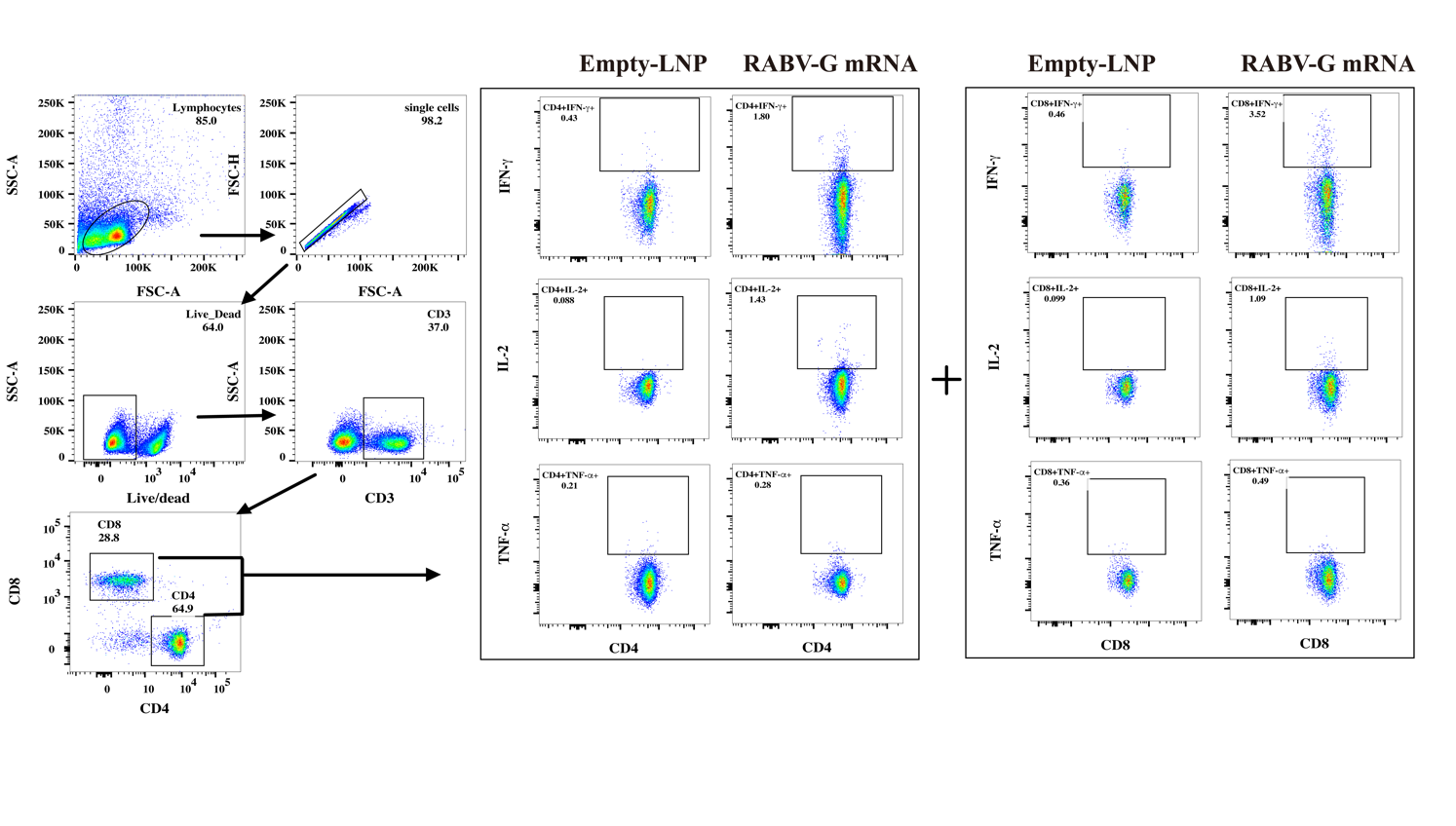


**Table S1 RABV-G peptides used in the study.**
